# Generation of induced neural stem cells with inducible IDH1^R132H^ for analysis of glioma development and drug testing

**DOI:** 10.1371/journal.pone.0239325

**Published:** 2020-09-18

**Authors:** Kamila Rosiak-Stec, Dagmara Grot, Piotr Rieske

**Affiliations:** Department of Tumor Biology, Medical University of Lodz, Lodz, Poland; Sechenov First Medical University, RUSSIAN FEDERATION

## Abstract

Mutation in isocitrate dehydrogenase 1 (IDH1^R132H^) occurs in various types of cancer, including low and high grade gliomas. Despite high incidence indicating its central role in tumor initiation and progression there are no targeted therapies directed against this oncogene available in the clinic. This is due to the limited understanding of the role of IDH1^R132H^ in carcinogenesis, which is further propagated by the lack of appropriate experimental models. Moreover, proper *in vitro* models for analysis of gliomagenesis are required. In this study, we employed a Tet On system to generate human induced neural stem cells with doxycycline-inducible IDH1^R132H^. Equivalent expression of both forms of IDH1 in the presented model remains similar to that described in tumor cells. Additional biochemical analyses further confirmed tightly controlled gene regulation at protein level. Formation of a functional mutant IDH1 enzyme was supported by the production of D-2-hydroxyglutarate (D2HG). All samples tested for MGMT promoter methylation status, including parental cells, proved to be partially methylated. Analysis of biological effect of IDH1^R132H^ revealed that cells positive for oncogene showed reduced differentation efficiency and viability. Inhibition of mutant IDH1 with selective inhibitor efficiently suppressed D2HG production as well as reversed the effect of mutant IDH1 protein on cell viability. In summary, our model constitutes a valuable platform for studies on the molecular basis and the cell of origin of IDH-mutant glioma (e.g. by editing P53 in these cells and their derivatives), as well as a reliable experimental model for drug testing.

## Introduction

The discovery of heterozygous mutations in genes encoding isocitrate dehydrogenase had a significant impact on our understanding of pathogenesis of gliomas and several other types of cancer, including hematologic malignancies, chondrosarcomas and cholangiocarcinomas [1]. The vast majority of mutations detected in gliomas are present in the cytosolic isoform IDH1, with substitution of arginine for histidine at codon 132 (IDH1^R132H^) accounting for 90% of all mutations in *IDH* genes [2]. In addition to the loss of normal function, the mutant protein acquires a neomorphic enzymatic activity resulting in NADPH-dependent reduction of α-ketoglutarate (αKG) to the oncometabolite D-2-hydroxyglutarate (D2HG) [3]. Accumulation of the latter may lead to various cellular dysfunctions and ultimately, to tumorigenesis. Numerous studies have confirmed several molecular mechanisms through which D2HG might exert its oncogenic effects, including competitive inhibition of enzymes that use αKG as a cofactor, such as chromatin modifying dioxygenases (Jmj family of histone demethylases and TET family of DNA demethylases) leading to altered histone and DNA methylation, inhibition of cell differentiation and malignant cell transformation [4–6]

Despite potential role of IDH1^R132H^ in tumor initiation and progression, its presence is linked to improved overall survival among glioma patients [7]. Nevertheless, due to high recurrence and progression rates of gliomas resulting in high mortality, targeted therapies against this oncogene are required. At present, no such therapies are available in the clinic. The most advanced drug candidates, including AG-120, AG-221 and AG-881 are in phase 1 clinical trials (NCT02073994; NCT02273739; NCT02481154) [8]. Those compounds were developed with intention of inhibiting the acquired catalytic activity of the mutant protein. However, alternative therapeutic strategies might be required to complement or substitute currently explored avenues of intervention. In addition to restoration of cell differentiation capabilities, attractive approaches involve targeting proteins acting as effectors of IDH1^R132H^ mutation or exploiting sensitivities ensuing from the aforementioned mutation [9–12].

To successfully employ such strategies, better understanding of the molecular mechanisms relevant to gliomagenesis is crucial. The challenge is further propagated by the scarcity of appropriate experimental models, since the culture of primary GB cells proved challenging to establish and maintain over an extended period of time [13–15]. Primary GB cells with IDH1 mutation have been reported to be even more susceptible to senescence, considered as one of the predominant causes of stabilization failure, compared to other primary GB cells [24, 25]. Only a few models with endogenous IDH1 mutation have been established, mainly orthotopic xenografts that constituted accompanying mutations [16]. On the other hand, expression of mutant IDH1 in mouse conditional knock-in models was reported to be lethal at an early stage of development or soon after transgene expression [17, 18]. Nevertheless, mouse models are not suitable for all studies, due to their limited throughput, bioethical issues, high costs, and variable time and rate of tumor formation, which may affect reproducibility of results. As a result, majority of studies on mutant IDH1 function have been performed on cell lines ectopically expressing this oncogene. These overexpression systems do not reflect the oncogenic properties of the heterozygous IDH1 mutation present in patient tumor cells [19] and do not offer the opportunity to analyze cellular origin of gliomas. Furthermore, distinct observations from experiments performed using these models have been reported (*i*.*e*. effect of mutant IDH1 protein on proliferation of U87 cell line [20–22]), possibly due to variations in cell lines between laboratories [23].

In this study we made an attempt to generate human induced neural stem cells with doxycycline-inducible IDH1^R132H^. Presented model captures all the aforementioned aspects, *i*.*a*. tightly regulated expression of the mutated gene, adequate ratio of wild type to mutant IDH1 protein, ability to differentiate into any neural lineage *in vitro*, and limited running costs. Further, we showed that expression of mutant IDH1 protein resulted in impaired differentiation and increased apoptosis.

## Materials and methods

### Cell culture

Induced Pluripotent Stem cells (iPSc) derived from human fibroblasts, obtained and characterized previously [24], were cultured on Geltrex coated dishes in Essential 8 medium (both from Life Technologies) at low oxygen conditions (5% O2/5% CO2).

Induced Neural Stem cells (iNSc) were generated from iPSc using PSC Neural Induction Medium (Gibco) according to the manufacturer’s protocol and propagated as an adherent culture on Geltrex coated dishes in Neural Expansion Medium (Neurobasal Medium: Advanced DMEM/F12 (1:1), supplemented with Neural Induction Supplement (1:50; all from Life Technologies). Cells were maintained at 37°C in humidified 5% CO2 incubator.

IDH-mutant glioma neurospheres generated from xenograft-forming tumor (MGG152) were a kind gift from Dr. Daniel P. Cahill, Massachusetts General Hospital [12, 16]. Neurospheres were cultured in DMEM/F12 medium supplemented with B27 (1:50; Life Technologies), human recombinant EGF (20 ng/mL; ABM), human recombinant basic FGF2 (10 ng/mL; ABM) and heparin (2 μg/mL; Sigma Aldrich). Cells were maintained at 37°C in humidified 5% CO2 incubator.

### Reagents

AGI-5198 was purchased from MedChemExpress. DMSO (solvent control) and doxycycline were purchased from Sigma-Aldrich.

### Genetic constructs and lentiviral vectors

The pLVX-EF1α-Tet3G transfer plasmid was purchased from Clontech. The pLVX-TRE3G-DEST was obtained as described previously [25]. The pLVX-TRE3G-GFP construct was obtained from the Department of Tumor Biology. The pLVX-TRE3G-IDH1^R132H^ construct was created by shuttling of IDH1^R132H^ sequence from the pENTR/Zeo-IDH1^R132H^ vector into pLVX-TRE3G-DEST with Gateway LR Clonase II (Life Technologies). To generate pENTR/Zeo-IDH1^R132H^, pENTR/Zeo-IDH1^WT^ vector (described previously [26]) was used for site-directed mutagenesis using Herculase II Fusion DNA Polymerase (Agilent). Primers containing the IDH1-G395A mutation were: CTATCATCATAGGTCATCATGCTTATGGGGATC (forward) and GATCCCCATAAGCATGATGACCTATGATGATAG (reverse). Following successful construction, confirmed by restriction enzyme digestion and direct sequencing, lentiviral vectors were prepared using LENTI-Smart (InvivoGen) according to the manufacturer’s protocol.

### Lentiviral transduction of induced neural stem cells

To establish induced neural stem cells expressing Tet-On transactivator gene, iNS cells were transduced with lentiviral particles prepared from pLVx-EF1α-Tet3G transfer plasmid (Clontech). After 48 hours, the cell culture medium was changed and the cells were selected by growth in G148 (200 μg/mL; Life Technologies). The selection medium was renewed every 2–3 days until no live cells in non-transduced control were observed. Subsequently, iNS cells with stably integrated Tet-On 3G regulatory gene were transduced with lentiviral vector carrying GFP or IDH1^R132H^ under the control of doxycycline-regulated TRE3GV promoter. After 48 hours, the cell culture medium was changed and the cells were selected with puromycin (0.5 μg/mL; InvivoGen). Pooled populations of puromycin resistant cells were obtained.

### Western blotting analysis

Cells were lysed in RIPA buffer supplemented with Protease Inhibitor Cocktail (both from Sigma Aldrich). Proteins were separated on 10% SDS–PAGE and transferred to the PVDF membrane (Immobilon—P, Merck Millipore) by electroblotting. Subsequently, membrane was blocked with 5% Skim Milk (Sigma Aldrich) and incubated at 4°C overnight with primary antibodies: anti-IDH1wt (1:1000, D2H1, Cell Signaling), anti-IDH1^R132H^ (1:100, H09, Dianova), anti-Actin (1:4000, MAB1501, Millipore). After washing, membrane was probed with appropriate HRP-conjugated secondary antibodies: anti-rabbit (1:4000, sc-2004, Santa Cruz Biotechnology), anti-mouse (1:4000, sc-2005, Santa Cruz Biotechnology). Bands were visualized with enhanced chemiluminescence (Amersham ECL Prime Western Blotting Detection Reagent, GE Healthcare) on ChemiDoc XRS (Bio-Rad). Densitometric analysis was performed using Image J software.

### Immunofluorescence analysis

For the immunocytochemical analyses induced pluripotent stem cells, induced neural stem cells and MGG152 neuropheres were seeded on Geltrex or poly-l-ornithine/laminin (both from Sigma Aldrich) coated 4-well plates, respectively. Cells were fixed in 4% paraformaldehyde in PBS for 15 min and permeabilized with 0.1% Triton X-100 in PBS for 10 minutes at room temperature. Non-specific binding sites were blocked with 2% donkey serum (Sigma Aldrich). Cells were subsequently incubated overnight with appropriate primary antibodies: anti-IDH1wt (1:800, D2H1, Cell Signaling), anti-IDH1^R132H^ (1:20, H09, Dianova), anti-Map2 (1:100, sc-7442, SantaCruz Biotechnology; 1:500, ab32454, Abcam), anti-GFAP (1:1000, ab7260, Abcam). After washing, cells were incubated with appropriate secondary antibodies for 1 hour at room temperature: anti-rabbit Alexa Fluor 488, anti-mouse Alexa Fluor 594 (1:500, both Life Technologies). The slides were mounted with ProLong Gold Antifade Reagent with DAPI (Molecular Probes), coverslipped, and imaged on fluorescent microscope (MN-800 FL, OPTA-TECH, Poland).

To quantify the GFP expression per cell following treatment with doxycycline, average GFP intensity was measured for five separate fields of view. Background was manually determined as no-cell-area and fluorescent was measured for it using ImageJ. Following subtraction of background, GFP intensity was divided by the number of cells (at least 200 per FOV) determined by DAPI counterstain, to determine intensity per cell.

### RNA isolation and quantitative Real-time PCR

Total RNA was isolated using AllPrep DNA/RNA Mini Kit (Qiagen). 250 ng of total RNA was used for reverse transcription using a QuantiTect Reverse Transcription Kit (Qiagen) according to the manufacturer’s protocol. Quantitative Real-time PCR was performed using StepOnePlus Real-Time PCR System (Applied Biosystems), as described previously [26]. Specific primers were used for amplification of the tested genes (Table 1). Normalized relative expression level was calculated using the method described previously by Pfaffl *et al*. [27].

**Table 1:** Primers used for quantitative Real-time PCR.

Gene	Forward primer (5’ → 3’)	Reverse primer (5’ → 3’)
*IDH1*	ACATGGTGGCCCAAGCTA	AGCAATGGGATTGGTGGA
*MGMT*	GCTGAATGCCTATTTCCACCA	CACAACCTTCAGCAGCTTCCA
*MEGF10*	TGACTGCTTGCCTGGCTTCACA	GTTACAGGTTCCGTTGTTGGTGC
*RASSF1A*	TGGGAGACACCTGACCTTTC	TGGGCAGGTAAAAGGAAGTG
*HEYL*	GAGAAACAGGGCTCTTCCAA	CTTCAAGGACCCCCAGGTA
*TNC*	TCACCAACTGTGCTCTGTCC	GCCTGCCTTCAAGATTTCTG
*TBP*	GAGCTGTGATGTGAAGTTTCC	TCTGGGTTTGATCATTCTGTAG

### Sanger sequencing

Products of reverse transcription reaction were used as a template for PCR amplification using primers nested in the exon 4 of *IDH1* gene using primers TCAGTGGCGGTTCTGTGGTA (forward), CCATGTCGTCGATGAGCCTA (reverse). RNA extracted from surgical tumor sample positive for *IDH1* mutation was obtained from the Department of Tumor Biology and used as a positive control. BigDye Seq kit v3.1 (Applied Biosystems) was used for labelling with following primers CATAGAGAATCGTGATGCCACC (forward), TTGGTGCTCAGA TACAAAGGC (reverse). The PCR-sequencing products were separated and analyzed using the ABI 3130xl Genetic Analyzer (Applied Biosystems). The studies were carried out in the Central Scientific Laboratory of the Medical University of Lodz.

### D-2HG content

The D-2HG level was measured by using a D2-HG assay kit (Abcam) according to the manufacturer’s protocol. The absorbance was recorded using microplate reader (Synergy 2, Biotek). Results were expressed as fold changes over the values for the controls.

### Cell viability

Cell viability was assessed using a CellTiter-Glo 2.0 kit (Promega) according to the manufacturer’s protocol. The luminescence was recorded using microplate reader (Synergy 2, Biotek). Results were expressed as fold changes over the values for the controls.

### Cell apoptosis

The activity of caspase-3/7 was determined using synthetic Caspase 3/7 activity reporter (CellEvent^™^ Caspase 3/7 Green; Life Technologies). After 72 hours of incubation with or without doxycycline, cells were co-incubated with the reporter for additional 24 hours, according to manufacturer’s instruction. Images were acquired using JuLI FL analyzer.

### MGMT gene promoter methylation

The *MGMT* promoter methylation status was analysed using Methylation-Specific PCR (MSP) and quantitative real-time Methylation-Specific PCR (qMSP). Genomic DNA was isolated using AllPrep DNA/RNA Mini Kit (Qiagen) and modified by sodium bisulfite treatment using the CiTi Converter DNA Methylation Kit (A&A Biotechnology) according to the manufacturers’ protocols. This DNA was afterwards used as template for MSP and qMSP. For each PCR reaction methylated DNA (Methylated Human Control, Promega) was used as positive control, and DNA from blood of healthy individual was used as negative control. Sample without DNA (water) was used as no template control.

MSP was carried out as described elsewhere [28, 29]. Two microliters of bisulfite-treated DNA was used for PCR with primers specific to either modified or unmodified DNA. The primer sequences were as follows: TTTCGACGTTCGTAGGTTTTCGC (methylated forward), GCACTCTTCCGAAAACGAAACG (methylated reverse), TTTGTGTTTTGATG TTTGTAGGTTTTTGT (unmethylated forward), AACTCCACACTCTTCCAAAAACAAA ACA (unmethylated reverse). The annealing temperature was 59°C. The 81 bp and 93 bp PCR products were separated by electrophoresis on 2% agarose gels and visualized. A sample was considered methylated when a band was observed in PCR with methylated primers.

Quantitative MSP was carried out as described by Pinson *et al*. [30]. Forward and reverse primers for *MGMT* were the same ones as used for MSP. The β-actin was used as a control for normalization. The primers used for ACTB were TAGGGAGTATATAGGTTGGGAAGTT (forward) and AACACACAATAACAAACACAAATTCAC (reverse). The relative level of methylated DNA was determined as a ratio of *MGMT* to *ACTB* multiplied by 1000.

### Statistical analysis

All experiments were performed three times, unless indicated otherwise. Statistical analysis was performed with GraphPad Prism 5 (Graphpad Software). The tests performed for each experiment are named in the figure legends for each experiment individually. The results were presented as the mean ± SEM. p values of 0.05 or less was considered statistically significant.

## Results

### Generation and characterization of inducible iNS cell line

**Fig 1:**
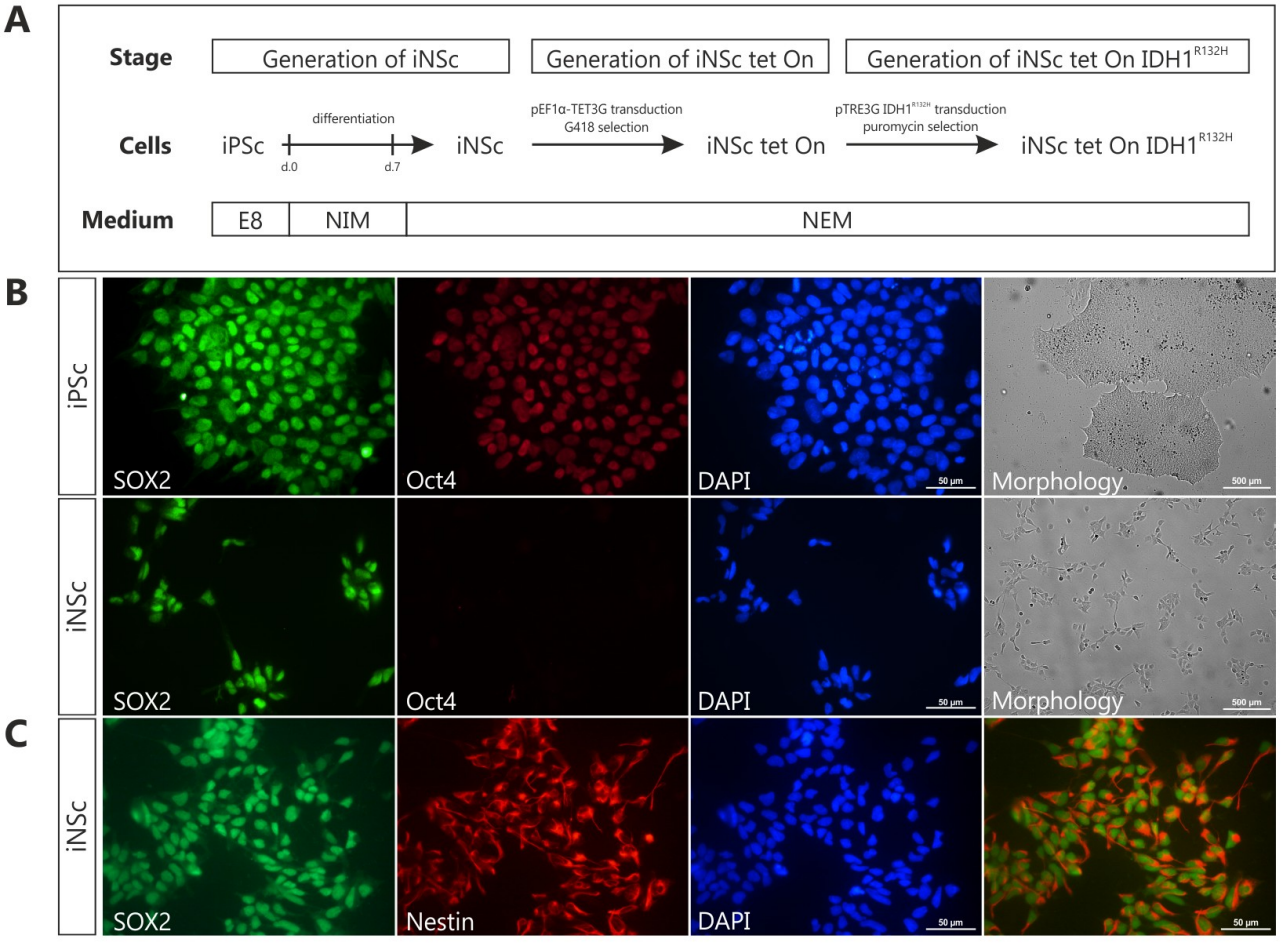
Generation of induced neural stem cells. **(A)** Schematic overview of the establishment of induced neural stem cells with doxycycline-inducible IDH1^R132H^. *E8 –Essential 8 medium*, *NIM—neural induction medium*, *NEM—neural expansion medium*. **(B)** Representative images showing iPSc and iPSc-derived iNSc immunostained with pluripotency markers SOX2 (*green*) and Oct4 (*red*), and morphology of the cell cultures under self-renewal conditions. Scale bars represent 50 and 500 μm (immunofluorescence and bright field images, respectively). **(C)** Staining of iNSc with neural stem cell markers SOX2 (*green*) and nestin (*red*). Cell nuclei were counterstained with the nuclear DNA marker DAPI (*blue*). Scale bars represent 50 μm.

Despite the cell origin of gliomas remains controversial, it is suggested that gliomas can originate from the neural stem cells [31, 32]. At the same time, establishing patient-derived cell cultures harboring *IDH1* mutation proved extremely challenging, as we reported previously [13]. Therefore, we have created induced neural stem cell line with doxycycline-regulated expression of IDH1^R132H^ (Fig 1A). By using serum-free neural induction medium, we first generated iPSc-derived induced neural stem cells (iNSc). To confirm that obtained cells differentiated successfully, we examined the expression of pluripotent marker, Oct4, and common NSC markers, SOX2 and Nestin. In contrast to iPSc, no Oct4-positive cells were observed in the iNSc culture (Fig 1B). Furthermore, all iNS cells were positive for SOX2 and Nestin, presenting the characteristic bipolar NSC morphology (Fig 1C).

**Fig 2:**
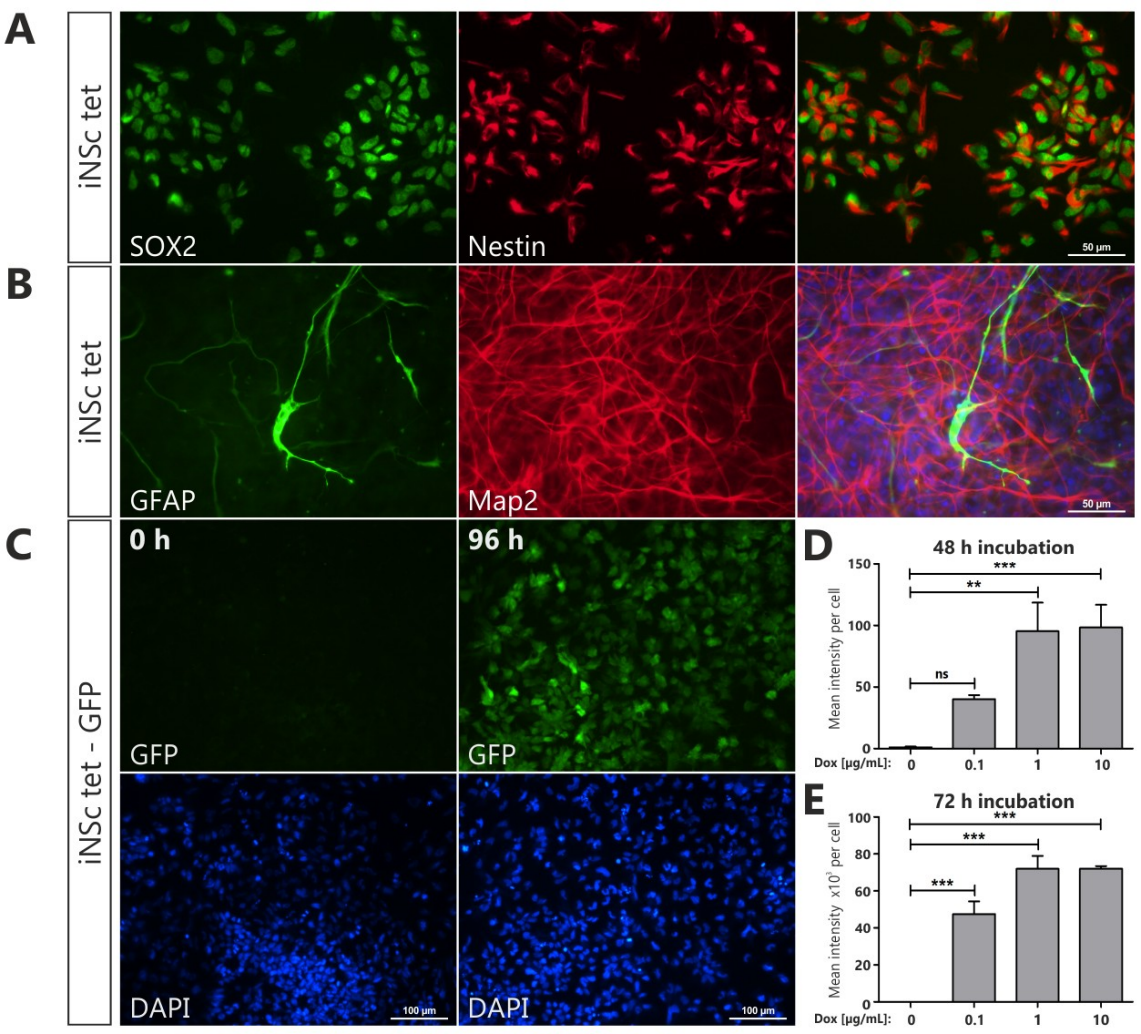
Stabilization of induced neural stem cells with tet-On transactivator gene. Representative images of **(A)** undifferentiated iNSc tet expressing neural precursor markers SOX2 (*green*) and nestin (*red*), and **(B)** differentiated iNSc tet expressing astroglia marker GFAP (*green*) and neuronal-specific marker Map2 (*red*). Cell nuclei were counterstained with the nuclear DNA marker DAPI (*blue*). Scale bars represent 50 μm (A-B). **(C)** Induction of green fluorescent protein (GFP, *green*) expression in iNSc tet-GFP treated with doxycycline (1 μg/mL, *Dox*) for 96 hours. Cell nuclei were counterstained with the nuclear DNA marker DAPI (*blue*). Scale bars represent 100 μm. **(D**, **E)** Quantification of GFP fluorescence intensity in iNSc tet-GFP after 48 and 72 hours of incubation with doxycycline at different concentrations. Statistical significance calculated by One-way ANOVA with Dunnett’s multiple comparison test. Error bars indicate SEM. **, p<0.01; ***, p<0.005; ns, not significant.

To generate inducible cell line, iNS cells were transduced with lentiviral vector carrying Tet-On 3G transactivation gene and were selected with G418 (Fig 1A). Transduced cells did not differ from the non-transduced ones and showed protein expression of NSC markers, SOX2 and Nestin, as confirmed immunocytochemically (Fig 2A). Obtained iNS tet cells were evaluated for their potential to differentiate into astrocytes and neurons. After culturing iNSc tet for three weeks in differentiation medium (Neurobasal Medium supplemented with B-27), the majority of cells developed into Map2-positive neurons with extensive neurites (Fig 2B). In contrast, only a small fraction of iNSc tet expressed the astrocyte marker, GFAP. To measure the response and activity of the inducible system, we stabilized cells engineered to express green fluorescent protein (GFP) upon doxycycline (Dox) treatment (Fig 2C). Induction time and doxycycline concentration were optimized using the aforementioned cells, with no GFP detected intrinsically (Fig 2C–2E). As expected, fluorescent signal was observed already after 24 hours of treatment and increased with time. Doxycycline concentration was tested in the range 0.1 to 10 μg/mL for all time points, with fluorescence reaching plateau at 1 μg/mL (Fig 2D and 2E). Taken together, those results support tightly controlled gene regulation with no ‘leakiness’ commonly associated with inducible systems. For the rest of the study, we chose the doxycycline dose 1 μg/mL and incubation time of 96 hours.

### Stabilization of induced neural stem cells inducibly expressing IDH1^R132H^

**Fig 3:**
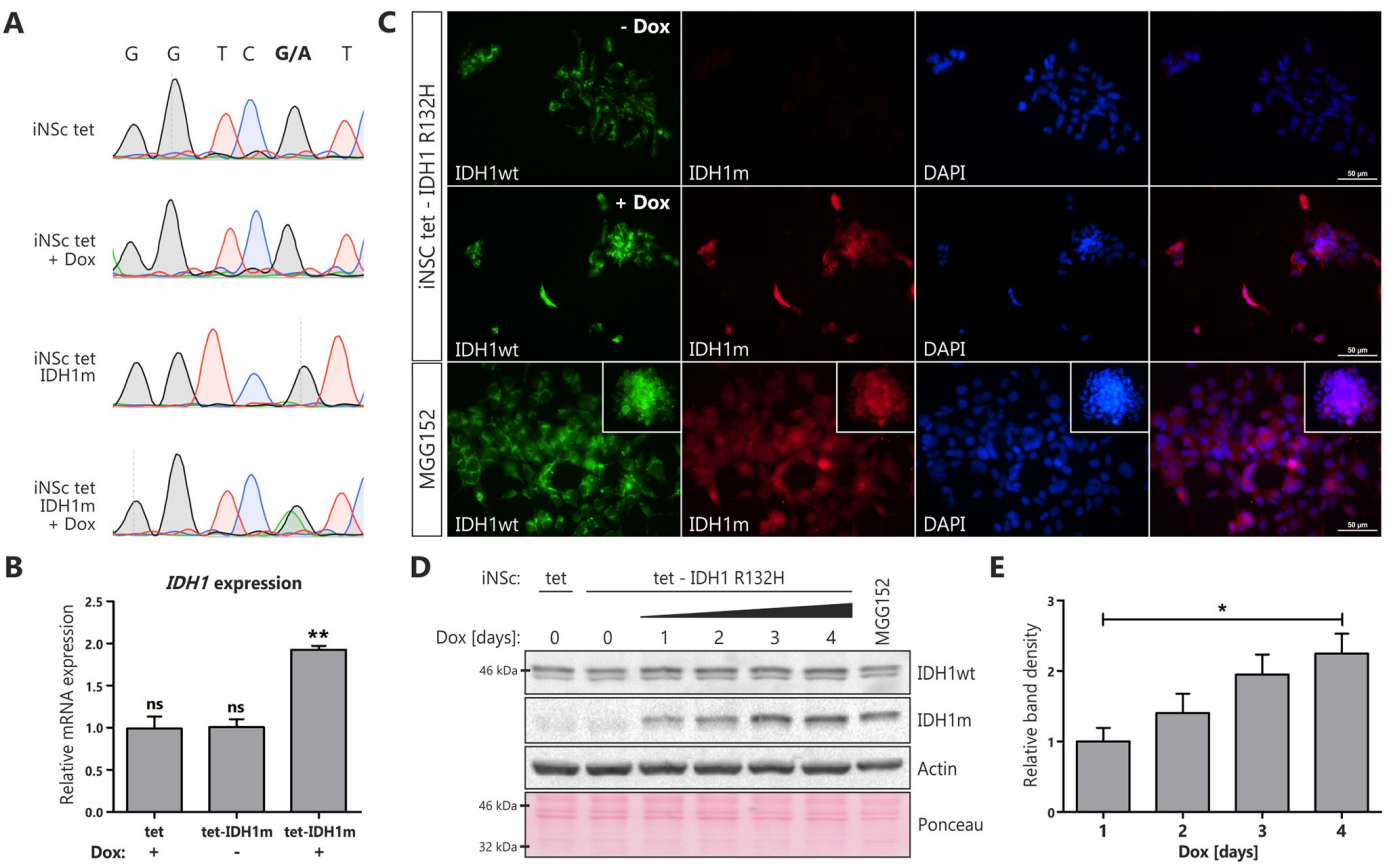
Characterization of induced neural stem cells with doxycycline-inducible IDH1^R132H^. Induced expression of IDH1^R132H^ upon doxycycline treatment. **(A)** Sanger sequencing electropherograms of the mutated nucleotide in codon 132 (R132H) in iNSc tet and iNSc tet-IDH1^R132H^ in the presence or absence of doxycycline (1 μg/mL, 96 h, *Dox*). **(B)** *IDH1* (non-mutation-specific) mRNA expression in iNSc tet and iNSc tet-IDH1^R132H^ stimulated or not with doxycycline (1 μg/mL, 96 h). Asterisks over bars indicate statistical significance towards untreated iNSc tet (not shown). Statistical significance calculated by One-way ANOVA with Dunnett’s multiple comparison test. Error bars indicate SEM, n = 3. **, p<0.01; ns, not significant. **(C)** Representative images of IDH1^R132H^ (*red*, *IDH1m*) and IDH1^WT^ (*green*) expression in iNSc tet-IDH1^R132H^ treated with doxycycline (1 μg/mL, 96 h) and MGG152 neurospheres with endogenous IDH1^R132H^. Cell nuclei were counterstained with the nuclear DNA marker DAPI (*blue*). Inset boxes show lower magnifications of MGG152 neurospheres. Scale bars represent 50 μm. **(D)** Western blot analysis of IDH1^R132H^ and IDH1^WT^ expression in iNSc tet, iNSc tet-IDH1^R132H^ untreated or treated with doxycycline (1 μg/mL) for indicated periods of time, and MGG152 neurospheres. Equal loading was verified by Ponceau S staining of the membrane. **(E)** Quantification of blot in D displaying ratio of band density of IDH1^R132H^ over Actin. Statistical significance calculated by One-way ANOVA with Dunnett’s multiple comparison test. Error bars indicate SEM, n = 3. *, p<0.05; ns, not significant.

To generate cells in which IDH1^R132H^ is expressed from a doxycycline-inducible cassette, we transduced iNS tet cells with lentiviral vector containing the cDNA sequence of IDH1^R132H^ under the control of a TRE3G promoter in a manner reminiscent of previously described GFP control. The presence of *IDH1* mutation was verified by amplifying cDNAs obtained by reverse transcription from isolated mRNAs, followed by Sanger sequencing (Fig 3A). Only engineered IDH1^R132H^ cells stimulated with doxycycline showed nucleotide alteration at position 395 (CGT to CAT) in codon 132. Peak height was reminiscent of that observed in the surgical tumor samples positive for *IDH1* mutation, suggesting expression to be at relevant physiological levels (S1 Fig). Of note, no mutant sequencing read was found in iNSc tet-IDH1^R132H^ in the absence of doxycycline. The RT-PCR analysis using primers that do not discriminate wild type from mutant *IDH1* confirmed approximately two-fold increase in transgene expression in iNS tet-IDH1^R132H^ cells stimulated with doxycycline (Fig 3B). Both results, two-fold increase in the mRNA expression and similar peak heights in sequencing electropherograms suggest equivalent expression of both forms of IDH1. We further examined transgene protein expression by immunostaining with an antibody specific to IDH1^R132H^ (Fig 3C). As expected, untreated iNS tet-IDH1^R132H^ cells lacked the mutant IDH1. In contrast, cells treated with doxycycline exhibited strong expression of IDH1^R132H^ comparable to expression of mutant IDH1 found in MGG152 neurospheres at endogenous level. Furthermore, expression of wild type IDH1 was comparable in all analyzed cell lines (Fig 3C and 3D). Additional biochemical analyses further confirmed the expression of mutant protein in doxycycline-treated iNS IDH1^R132H^ cells, but not in control cells (Fig 3D). Incubation of iNSc tet-IDH1^R132H^ with doxycycline for indicated periods of time showed that the effect of doxycycline on protein expression was time-dependent (Fig 3D, quantified in 3E).

### iNSc tet-IDH1^R132H^ as experimental mode*l*

**Fig 4:**
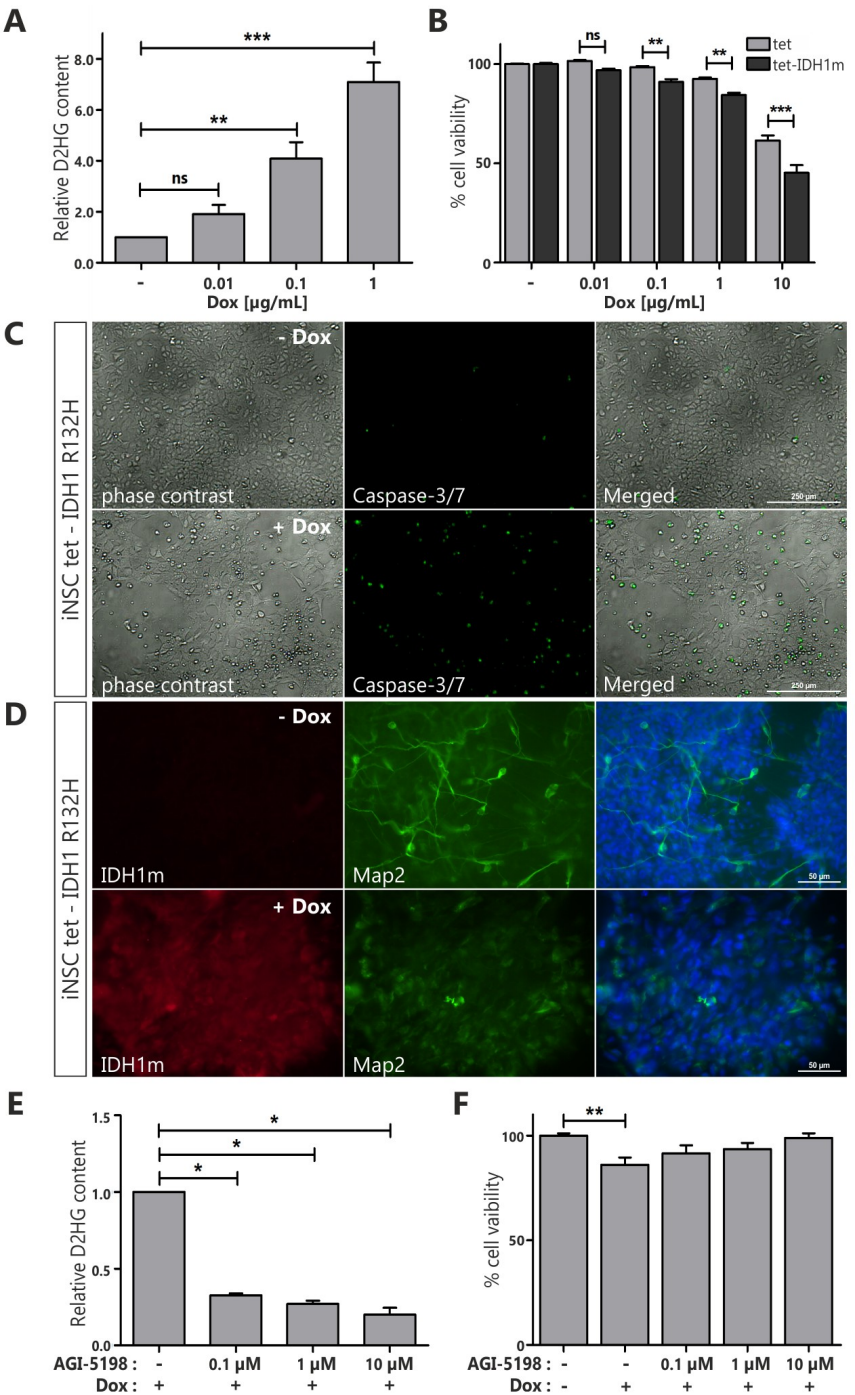
Evaluation of iNSc tet-IDH1^R132H^ as experimental model for drug testing. **(A)** Relative D2HG content in iNSc tet-IDH1^R132H^ treated with indicated concentrations of doxycycline for 96 hours. Statistical significance calculated by One-way ANOVA with Dunnett’s multiple comparison test. **(B)** Cell viability at day 6 in iNSc tet and iNSc tet- IDH1^R132H^ untreated or treated with doxycycline at the indicated concentrations. Statistical significance calculated by Two-way ANOVA with Bonferroni’s post-comparison test. **(C)** Representative images showing activity of the synthetic Caspase 3/7 reporter in iNSc tet-IDH1^R132H^ untreated or treated with doxycycline (1 μg/mL) for 96 hours. For better readability, the light microscopy images were contrast-enhanced. Scale bars represent 250 μm **(D)** Representative images of immunocytochemical characterization of Map2 (*green*) and IDH1^R132H^ (*red*, *IDH1m*) expression after 14 days of differentiation in iNSc tet-IDH1^R132H^ untreated or treated with doxycycline (1 μg/mL). Cell nuclei were counterstained with the nuclear DNA marker DAPI (*blue*). Scale bars represent 50 μm. **(E)** Relative D2HG content in iNSc tet-IDH1^R132H^ treated with doxycycline (1 μg/mL) and IDH1^R132H^-specific inhibitor AGI-5198 at the indicated doses for 96 hours. Statistical significance calculated by One sample *t* test, n = 2. **(F)** Cell viability at day 4 in iNSc tet- IDH1^R132H^ treated or not with doxycycline (1 μg/mL) and IDH1^R132H^-specific inhibitor AGI-5198 at the indicated doses (normalized to DMSO control). Statistical significance calculated by One-way ANOVA with Dunnett’s multiple comparison test. Error bars indicate SEM, n = 3. *, p<0.05; **, p<0.01; ***, p<0.005; ns, not significant.

Mutant IDH1 protein acquires new enzymatic activity leading to the production of D-2-hydroxyglutarate, which is considered a major driver of tumorigenesis [3, 4]. To test functionally the established iNSc tet-IDH1^R132H^, we measured the D2HG concentrations in cells treated for 96 hours with doxycycline at various concentrations (0.1–1 μg/mL) or in control cells. D2HG levels were elevated by 2–7 fold upon doxycycline administration and correlated with doxycycline concentration (Fig 4A). Since D2HG has been reported as a major intracellular effector of mutant IDH1 phenotype, inducing DNA hypermethylation and G-CIMP [33], we further assessed the methylation status of the *MGMT* gene promoter (S2 Fig). Engineered iNS tet-IDH1^R132H^ cells were treated with doxycycline (1 μg/mL) for 4 or 14 days followed by genomic DNA isolation and sodium bisulfite modification. Non-quantitative MSP showed that the *MGMT* promoter proved to be partially methylated in all samples tested, including parental cells (S2A Fig), suggesting that promoter methylation might originate in cell culture as an adaptation mechanism or as an effect of cellular stemness manipulation. Further analysis performed with quantitative MSP indicated that the methylation level of *MGMT* remains unchanged over time (S2B Fig). Finally, since it is suggested that hypermethylation caused by mutant IDH1 results in altered gene expression, usually their downregulation, we examined expression of additional genes described to be methylated in IDH1-mutant gliomas or in engineered IDH1-mutant cells [34–36]. Expression of *MEGF10* indicated a downward trend (p = 0.0525), however all genes tested did not display statistically significant difference in expression (S2C Fig).

We have previously reported increased IDH1^R132H^-dependent apoptosis in iNSc with constitutive expression of the oncogene of interest [26]. To determine the effects of regulated levels of IDH1 expression on cell viability, we treated iNS tet-IDH1^R132H^ cells with doxycycline concentrations ranging from 0.1 to 10.0 μg/mL for 6 days (Fig 4B). Although doxycycline itself significantly contributed to cell death at high concentration, cells with induced expression of IDH1^R132H^ demonstrated further reduction in cell viability at all concentrations tested. To elucidate the mechanisms causing decreased cell viability, we assessed caspase-3/7 activation as an indicator of apoptosis induction. After 96 hours of treatment with doxycycline (1 μg/mL), iNS tet-IDH1^R132H^ cells were incubated with a synthetic reporter of caspase 3/7 activity. Microscopic observations revealed more prominent caspase-3/7 activity in cells with IDH1^R132H^ expression (Fig 4C). This result indicates that mutant IDH1 protein induced apoptotic cell death.

Furthermore, it has been reported by us and other groups that the mutant IDH1 protein can lead to inhibition of differentiation. To assess the effect of IDH1^R132H^ expression on the differentiation efficiency of iNSc tet-IDH1^R132H^, cells were cultured in differentiation medium in the presence or absence of doxycycline (Fig 4D; with quantification of differentation phenotype in S3 Fig). Staining with antibody recognizing Map2, a neuronal lineage marker, revealed no Map2-positive cells with characteristic extensive neurites in cultures with induced expression of IDH1^R132H^ after two weeks of differentiation. This is in contrast to unstimulated iNSc tet-IDH1^R132H^. Additionally, doxycycline itself had no effect on differentiation of iNS tet cells (S4A Fig; with quantification of differentation phenotype in S4B Fig).

Finally, we used our functionally validated iNS tet-IDH1^R132H^ cell line to assess the activity of AGI-5198, enzymatic inhibitor selective for mutant IDH1. AGI-5198 efficiently suppressed D2HG production in a dose-dependent manner upon 96 hours of co-treatment with doxycycline (Fig 4E). Additionally, AGI-5198 treatment reversed very modest effect of mutant IDH1 protein on cell viability (Fig 4F). This effect can be attributed to the intended inhibitory function and not an off-target, as AGI-5198 treatment at the highest tested concentration had no impact on iNS tet cell lines viability (S5 Fig).

## Discussion

Glioblastoma (GB) is the most common and aggressive primary malignant brain tumor in adults. Genetic evidence supports the notion that IDH1 mutation plays a causal role in gliomagenesis. Further research aimed at elucidation of the molecular mechanisms underlying this process, thereby contributing to development of effective therapies against this modality are needed.

Considering the significant amount of evidence present in the literature indicating NSC as a candidate cell of origin of gliomas [31, 32, 37–39], we employed a Tet On system to generate human induced neural stem cells with doxycycline-inducible IDH1^R132H^. Established cell line retained its stem cell characteristics in the form of Sox2 and Nestin expression. Expression level of endogenous IDH1 protein was not altered as compared to control non-transduced iNS tet cells and was reminiscent of that in IDH1-mutant neurospheres generated from xenograft-forming tumor. Engineered IDH1^R132H^ cells showed dose- and time-dependent activation of mutant IDH1 expression after doxycycline treatment, with no ‘leakiness’ in the absence of doxycycline. This aspect is essential feature of the proposed model, as it allows for differentiation of engineered NSCs into any neural lineage including astrocytes and oligodendrocyte progenitor cells, prior to induction of IDH1^R132H^ expression. This approach allows further investigations on effects of mutant IDH1 on distinct candidate cells of origin and their role in gliomagenesis.

While on genetic level the number of *IDH1* wild-type and mutant copies is higher in our model than what is observed in clinical settings, the stoichiometry of transcripts as well as proteins remains similar to that described in patients. Considering the importance of stoichiometry and functional character of proteins, we propose that in this sense, our model reflects the molecular aspect of *IDH1*-mutant gliomas. Furthermore, using quantitative and non-quantitatve methods we have observed methylation of the *MGMT* gene promoter, a hallmark of IDH1 mutant gliomas [40]. However, we were unable to detect changes in promoter methylation within 14 days of mutant IDH1 induction, which might be caused by insufficient timeframe. It is important to point out that DNA methylation was detected in parental cells, making it challenging to draw direct correlation between the mutant IDH1 expression and promoter methylation.

Our findings on the effect of mutant IDH1 on cell differentiation corroborate previous reports [5, 26, 33, 41, 42]. We have observed reduced relative number of differentiated cells after induction of mutant IDH1 expression compared to the unstimulated or non-transduced cells. Negative effect of mutant IDH1 on differentiation has been reported and correlated with methylation changes induced by D2HG [5, 33]. In our model, we confirmed regulated formation of D2HG in cells expressing mutant IDH1. It is worth mentioning that impaired differentiation is a hallmark of cancer cells and restoration of cell differentiation capabilities holds great promise for cancer treatment [43–45]. In this study, we showed that AGI-5198, selective inhibitor of IDH1^R132H^, blocked the ability of the mutant enzyme to produce D2HG and reversed its negative effect on cell viability.

The pro-apoptotic effect of mutant IDH1 has been previously reported by us and others, underscoring the limited understanding of the tumorigenesis and the role played by IDH1 mutations therein [17, 26, 46–50]. Despite central character of mutant IDH1 protein in gliomagenesis, reports in the literature showed that mutant IDH1 promotes apoptosis by sensitizing glioma cells to ER stress through upregulation of miR-183 and suppression of its target Semaphorin E3, or by negatively regulating Wnt/β-catenin signaling [46, 47]. Our finding that mutant IDH1 triggered a mild increase in apoptosis of neural stem cells was corroborated by us here. Li and colleagues demonstrated that despite mutant IDH1 slightly increased apoptosis in U87MG cells it also triggered hypersuccinylation that imposes stresses to mitochondria and confers resistance to stress-induced apoptosis [48]. Studies by Modrek *et al*. showed that increased cell death induced by mutant IDH1 was rescued by P53 knockdown, postulating that only the combination of the 3 oncogenic hits, IDH1 mutation, followed by loss of P53 and loss of ATRX, promotes gliomagenesis [49]. Also Philip *et al*. reported that mutant IDH1 is not sufficient on its own to promote gliomagenesis *in vivo* in a mouse model and requires cooperation with other genetic alterations [51]. Taking into account the aforementioned findings, we propose that our model is a suitable tool that can be further altered to investigate interactions and interdependencies in a controlled and reproducible fashion with added benefit of choice of neural cell lineage, as discussed before. At the same time, it is important to note scarcity and limited availability of models for research focused on mutant IDH1 protein’s role in gliomagenesis. One potential avenue of future research utilizing the presented model is analysis of P53 editing in these cells or their derivatives.

Although no targeted small molecules that can inhibit mutant IDH1 protein have been approved for glioma therapy, a number of inhibitors are still in early clinical phases. It has been reported that these inhibitors are able to reduce 2-hydroxyglutarate level, enhance differentiation and inhibit tumor growth in patient-derived xenograft mouse models [44, 52–54]. However, since IDH1 mutation correlates with better prognosis and longer overall survival [7], administration of IDH1^R132H^ inhibitors with conjunction of temozolomide or radiotherapy remains a subject of debate. It is suggested that mutant IDH1 protein increases sensitivity for radiation and temozolomide through substantial NADPH depletion [6, 55, 56]. Molenaar *et al*. showed that coadministration of AGI-5198 inhibitor with radiotherapy resulted in limiting the effectiveness of irradiation [56]. In contrast, Nicolay *et al*. reported that coadministration of AG-881 with radiotherapy significantly reduced tumor growth than irradiation alone. Moreover, treatment with inhibitor did not affect temozolomide efficacy [53]. Those contradicting reports warrant necessity for in depth investigation of molecular mechanisms governing gliomagenesis and disease progression. One of the alternative approaches to treating mutant IDH1 gliomas involves targeting effectors of IDH1^R132H^ mutation or exploiting sensitivities ensuing from the aforementioned mutation [9–12]. Our model offers a viable platform for such studies as well as for subsequent drug-development campaigns.

In summary, this study provides a novel approach to model IDH1 mutant glioma that complements existing models. Generation of cells in which mutant IDH1 is expressed from a doxycycline-inducible cassette enables tight regulation of the oncogene’s expression in terms of its induction at adequate protein level and desired time point. Furthermore, mutant IDH1 expression can be induced following NSCs differentiation into alternative putative cells of origin of glioma. Additional modification can be introduced into the model to investigate selected aspects of genetic interactions. Finally, besides plasticity, this model is accessible and suitable for large scale studies, including applications in drug-development efforts.

## Supporting information

S1 File (PDF)

S1 Fig Detection of IDH1 mutation by Sanger sequencing. Representative electropherograms of the *IDH1* mutation in codon R132 in tumor specimens obtained from 3 patients diagnosed with IDH-mutant glioblastoma.
(TIF)

S2 Fig Methylation changes in iNSc expressing IDH1^R132H^. **(A)** Representative results of methylation specific PCR analysis of MGMT in parental cells and iNSc tet-IDH1^R132H^ treated with doxycycline (1 μg/mL) for 4 or 14 days. Bisulphite-modified DNA was amplified with primers specific for unmethylated (U) and methylated (M) DNA. **PC**: positive control, Methylated Human Control; **NC**: negative control, DNA obtained from blood sample; **NTC**: no templated control, water. **Lane L**: 50 bp DNA Ladder. **(B)** *MGMT* promoter methylation level in iNSc tet-IDH1^R132H^ stimulated with doxycycline (1 μg/mL) for 14 days. Statistical significance calculated by two-tailed Student’s t test. Error bars indicate SEM, n = 3. All changes are not significant. **(C)** Relative mRNA expression of indicated genes in iNSc tet-IDH1^R132H^ stimulated with doxycycline (1 μg/mL) for 14 days. Statistical significance calculated towards untreated iNSc tet-IDH1^R132H^ (not shown) by One sample t test. Error bars indicate SEM, n = 3. All changes are not significant.
(TIF)

S3 Fig Impaired neuronal differentiation of iNSc expressing IDH1^R132H^. Quantification of the differentiation phenotype, expressed as the ratio of Map2-positive cells with the elongated morphology in the population, after 14 days of differentiation in iNSc tet-IDH1^R132H^ untreated or treated with doxycycline (1 μg/mL). The proportion of positive cells was obtained by analyzing at least 200 cells per random field, with at least three fields taken per condition. Statistical significance calculated by two-tailed Student’s t test. Error bars indicate SEM. ***, p<0.005.
(TIF)

S4 Fig Effect of doxycycline treatment on iNSc tet differentiation. **(A)** Representative images of immunocytochemical staining of Map2 (*green*) expression after 14 days of differentiation in iNSc tet untreated or treated with doxycycline (1 μg/mL). Cell nuclei were counterstained with the nuclear DNA marker DAPI (*blue*). Scale bars represent 50 μm. **(B)** Quantification of the differentiation phenotype, expressed as the ratio of Map2-positive cells with the elongated morphology in the population, after 14 days of differentiation in iNSc tet untreated or treated with doxycycline (1 μg/mL). The proportion of positive cells was obtained by analyzing at least 200 cells per random field, with at least three fields taken per condition. Statistical significance calculated by two-tailed Student’s t test. Error bars indicate SEM. ns, not significant.
(TIF)

S5 Fig Effect of AGI-5198 treatment on iNSc tet viability. Cell viability at day 4 in iNSc tet treated with IDH1^R132H^-specific inhibitor AGI-5198 at the indicated doses (normalized to DMSO control). Statistical significance calculated by One-way ANOVA with Dunnett’s multiple comparison test. Error bars indicate SEM, n = 3. ns, not significant.
(TIF)
