# Supplementary material for: Generation of induced neural stem cells with inducible IDH1R132H for analysis of glioma development and drug testing
Source: PLoS One. 2020 Sep 18;15(9):e0239325. doi: 10.1371/journal.pone.0239325 (PMC7500637; doi:10.1371/journal.pone.0239325)

S1_raw_images

Figure 3D raw data
anti-IDH1wt antibody
Chemidoc

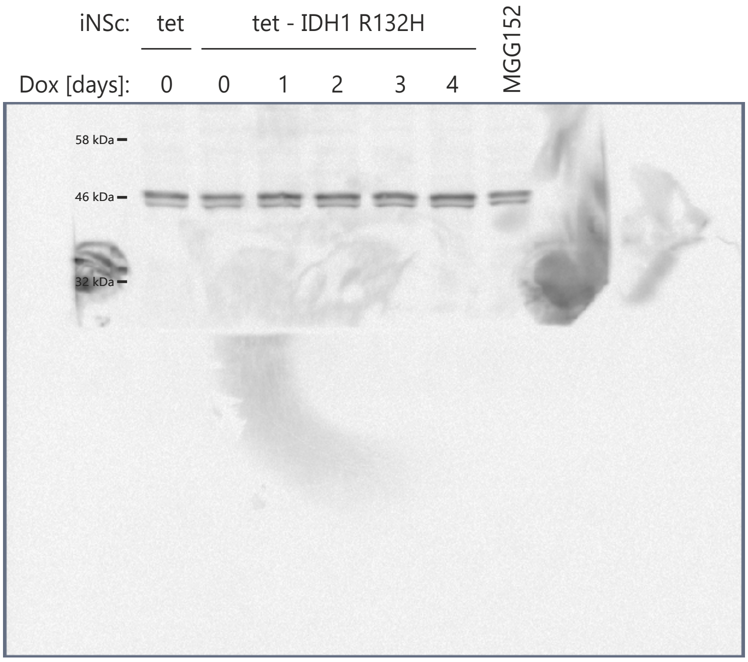

Figure 3D raw data
anti-IDH1R132H antibody
Chemidoc

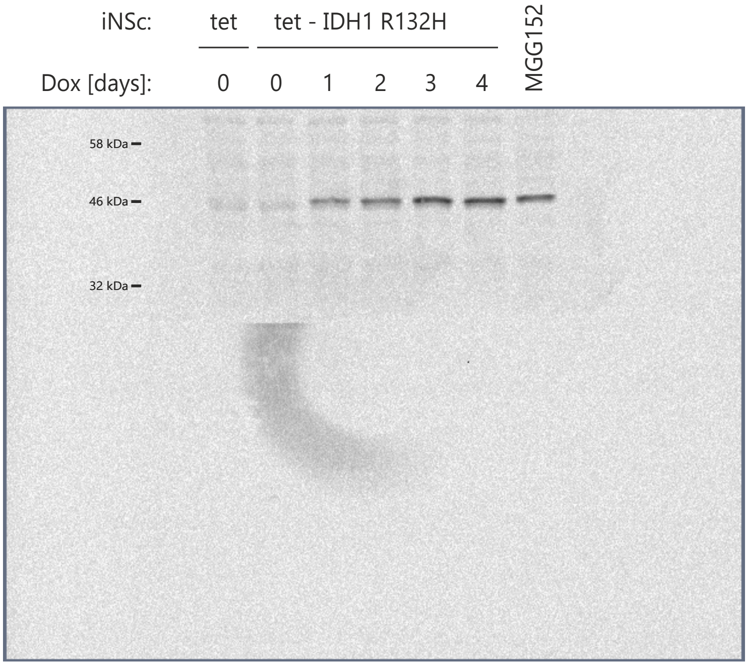

Figure 3D raw data
anti-Actin antibody
Chemidoc

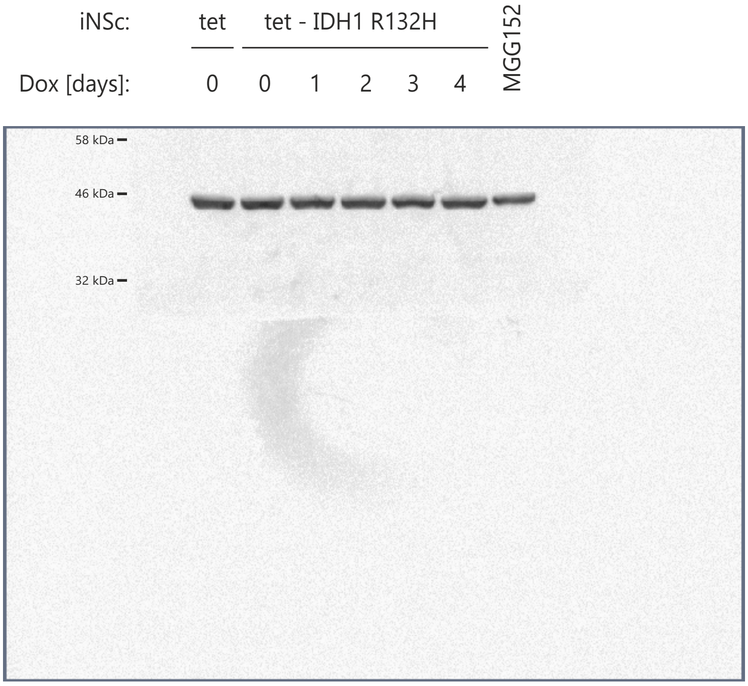

S1_raw_images

Supporting 1 Figure raw data

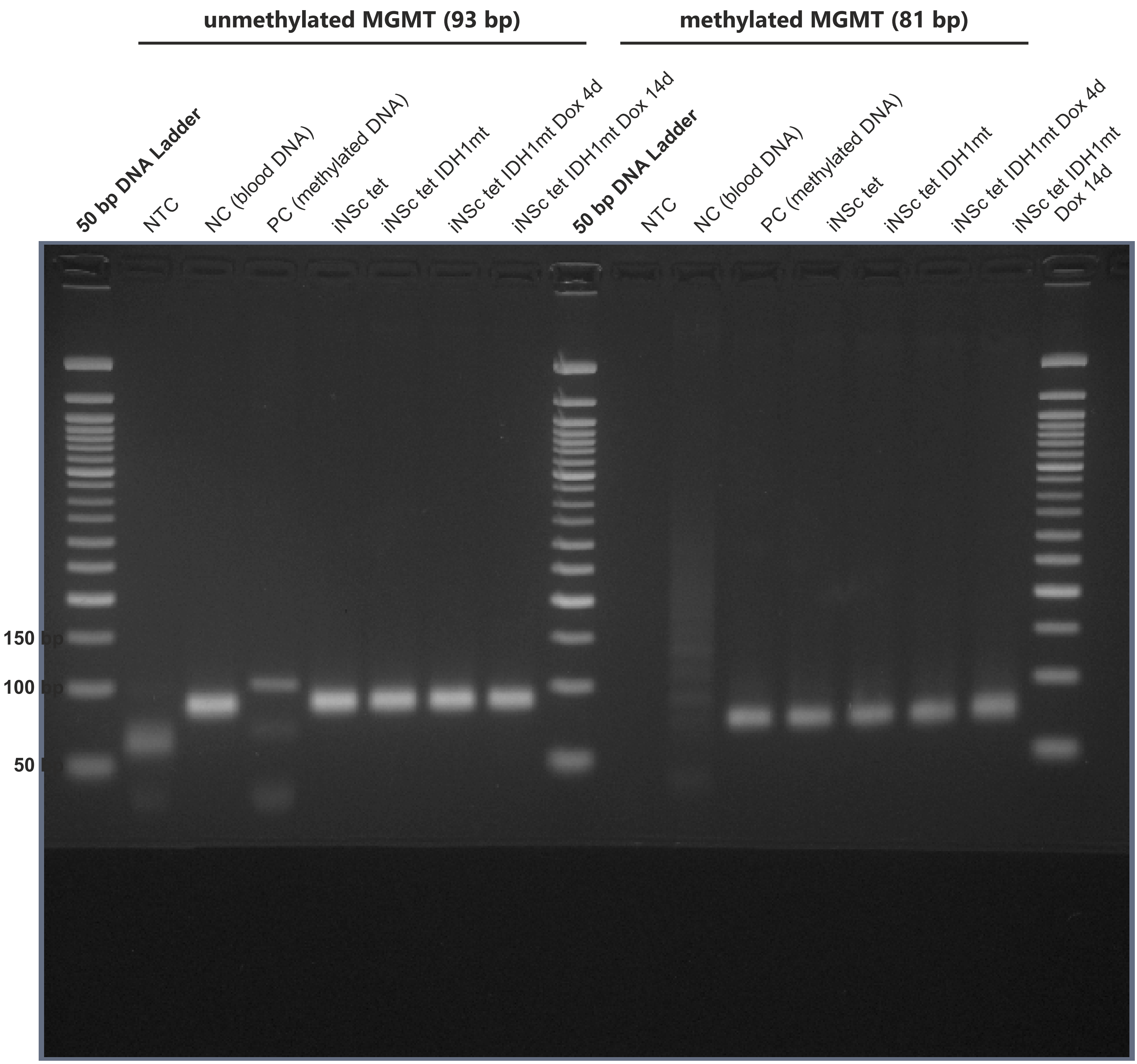

S1_raw_images

Supporting 1 Figure raw data

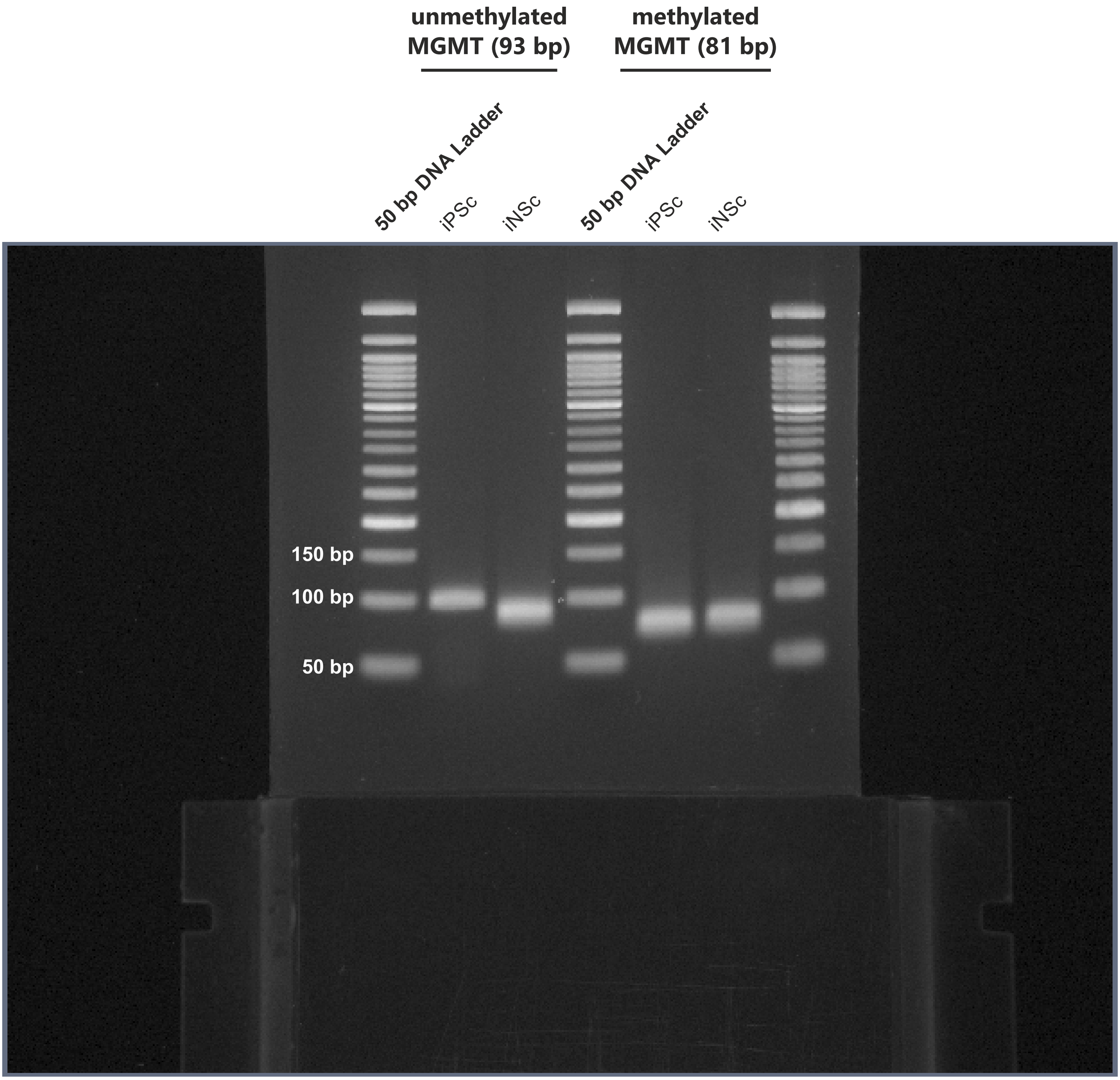

Supplement: S1 File — (PDF) [file pone.0239325.s001.pdf]
