# Supplementary figures and images for: Generation of induced neural stem cells with inducible IDH1R132H for analysis of glioma development and drug testing
Source: PLoS One. 2020 Sep 18;15(9):e0239325. doi: 10.1371/journal.pone.0239325 (PMC7500637; doi:10.1371/journal.pone.0239325)

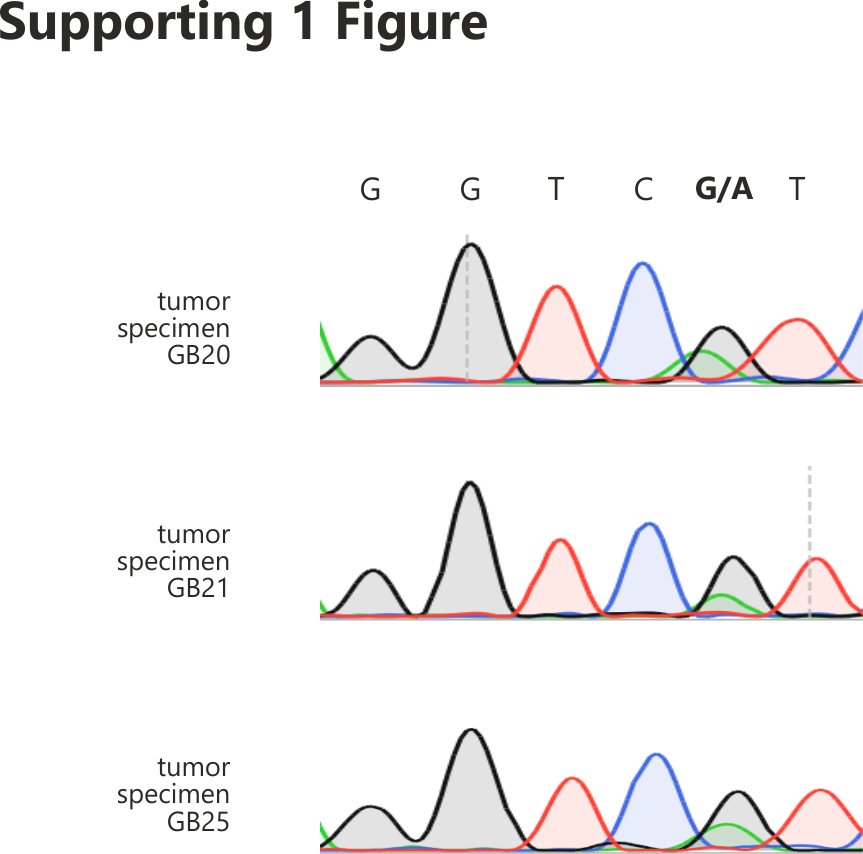

Supplement: S1 Fig — Representative electropherograms of the IDH1 mutation in codon R132 in tumor specimens obtained from 3 patients diagnosed with IDH-mutant glioblastoma. (TIF) [file pone.0239325.s002.tif]

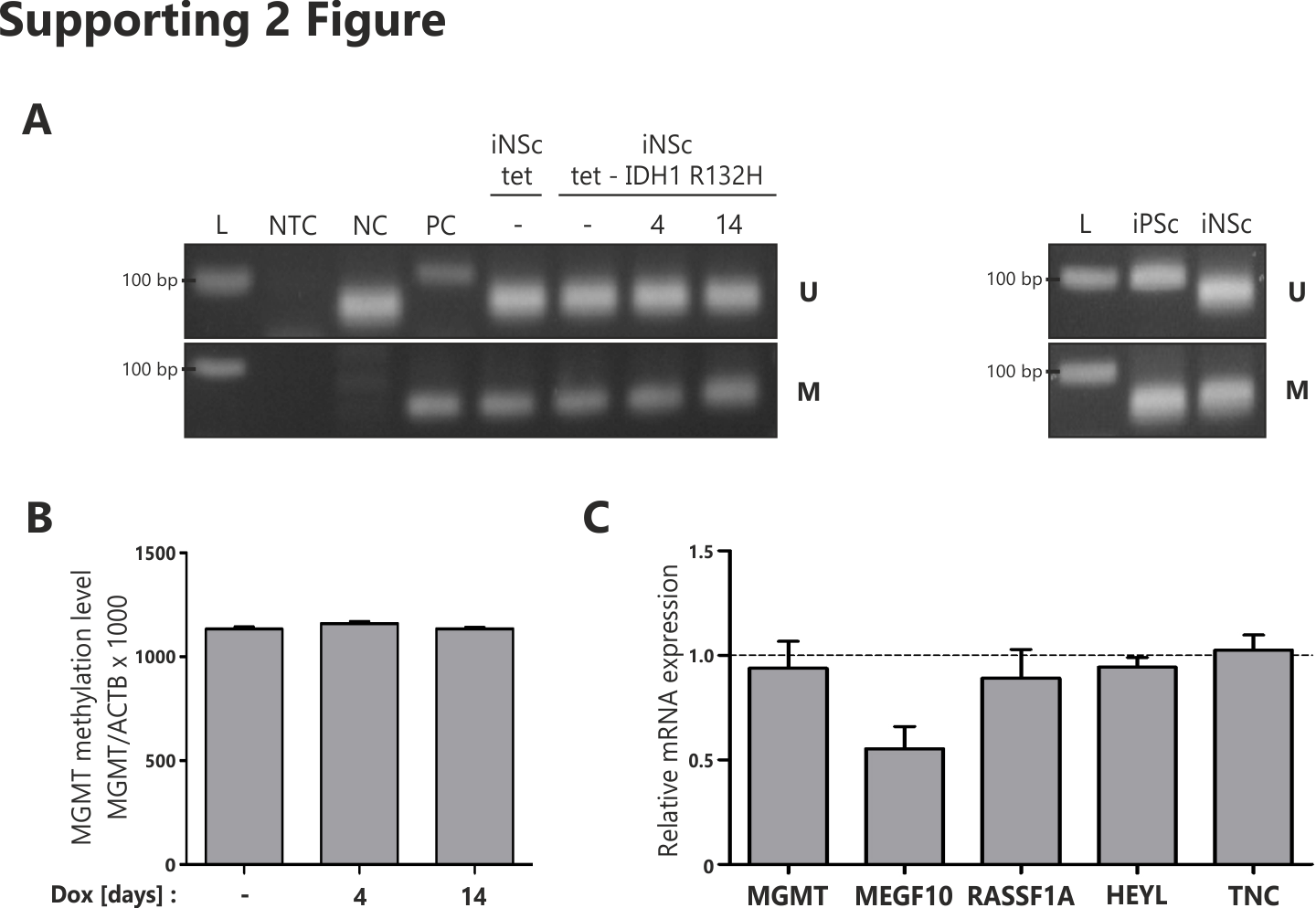

Supplement: S2 Fig — (A) Representative results of methylation specific PCR analysis of MGMT in parental cells and iNSc tet-IDH1R132H treated with doxycycline (1 μg/mL) for 4 or 14 days. Bisulphite-modified DNA was amplified with primers specific for unmethylated (U) and methylated (M) DNA. PC: positive control, Methylated Human Control; NC: negative control, DNA obtained from blood sample; NTC: no templated control, water. Lane L: 50 bp DNA Ladder. (B) MGMT promoter methylation level in iNSc tet-IDH1R132H stimulated with doxycycline (1 μg/mL) for 14 days. Statistical significance calculated by two-tailed Student’s t test. Error bars indicate SEM, n = 3. All changes are not significant. (C) Relative mRNA expression of indicated genes in iNSc tet-IDH1R132H stimulated with doxycycline (1 μg/mL) for 14 days. Statistical significance calculated towards untreated iNSc tet-IDH1R132H (not shown) by One sample t test. Error bars indicate SEM, n = 3. All changes are not significant. (TIF) [file pone.0239325.s003.tif]

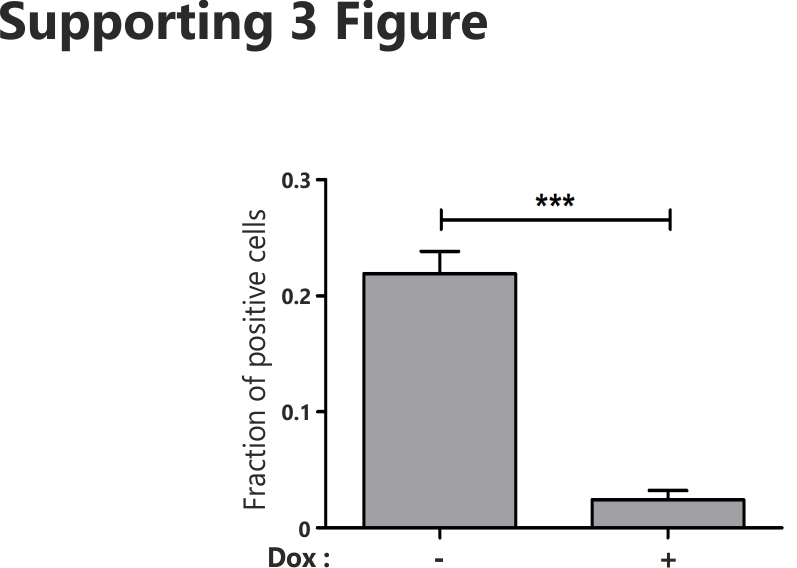

Supplement: S3 Fig — Quantification of the differentiation phenotype, expressed as the ratio of Map2-positive cells with the elongated morphology in the population, after 14 days of differentiation in iNSc tet-IDH1R132H untreated or treated with doxycycline (1 μg/mL). The proportion of positive cells was obtained by analyzing at least 200 cells per random field, with at least three fields taken per condition. Statistical significance calculated by two-tailed Student’s t test. Error bars indicate SEM. ***, p<0.005. (TIF) [file pone.0239325.s004.tif]

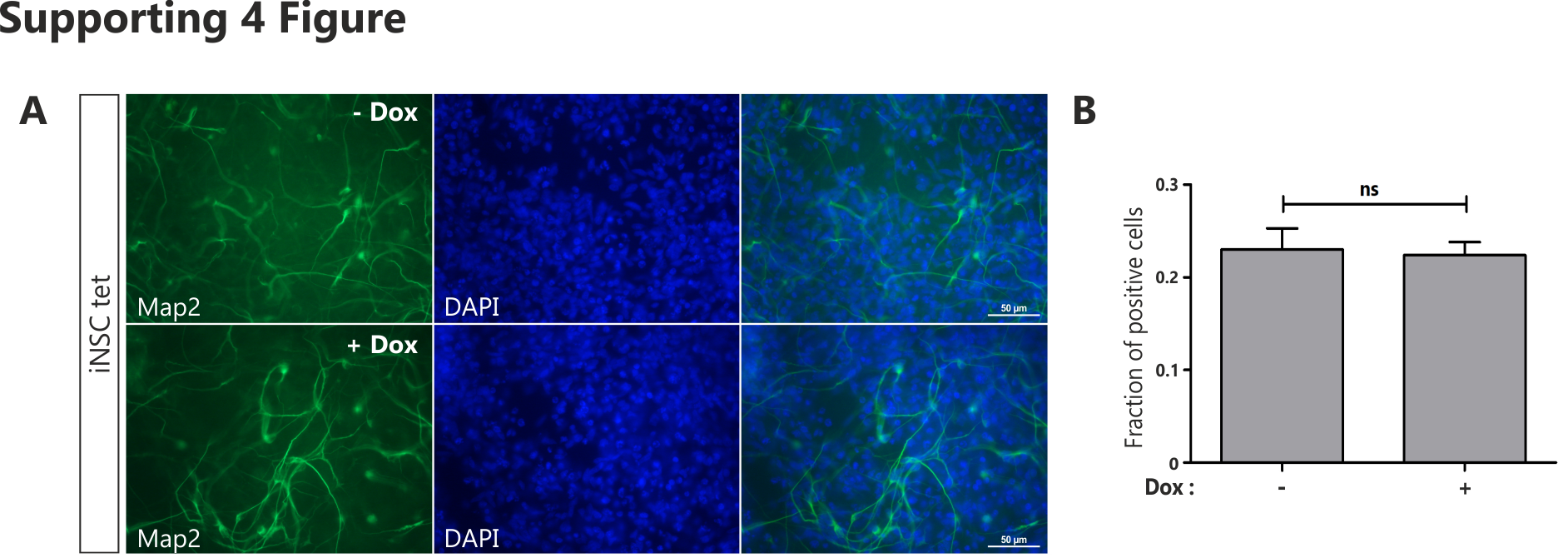

Supplement: S4 Fig — (A) Representative images of immunocytochemical staining of Map2 (green) expression after 14 days of differentiation in iNSc tet untreated or treated with doxycycline (1 μg/mL). Cell nuclei were counterstained with the nuclear DNA marker DAPI (blue). Scale bars represent 50 μm. (B) Quantification of the differentiation phenotype, expressed as the ratio of Map2-positive cells with the elongated morphology in the population, after 14 days of differentiation in iNSc tet untreated or treated with doxycycline (1 μg/mL). The proportion of positive cells was obtained by analyzing at least 200 cells per random field, with at least three fields taken per condition. Statistical significance calculated by two-tailed Student’s t test. Error bars indicate SEM. ns, not significant. (TIF) [file pone.0239325.s005.tif]

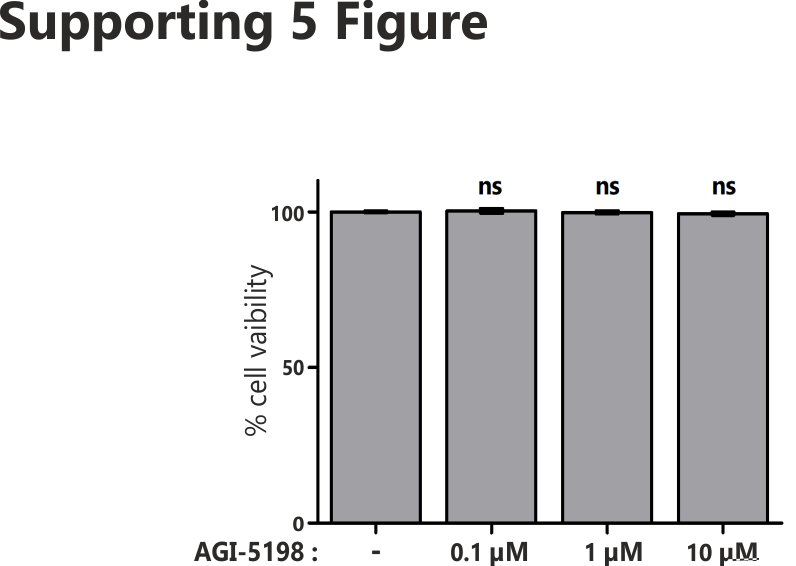

Supplement: S5 Fig — Cell viability at day 4 in iNSc tet treated with IDH1R132H-specific inhibitor AGI-5198 at the indicated doses (normalized to DMSO control). Statistical significance calculated by One-way ANOVA with Dunnett’s multiple comparison test. Error bars indicate SEM, n = 3. ns, not significant. (TIF) [file pone.0239325.s006.tif]
